# Optimizing Fluorescein Isothiocyanate Dextran Measurement As a Biomarker in a 24-h Feed Restriction Model to Induce Gut Permeability in Broiler Chickens

**DOI:** 10.3389/fvets.2017.00056

**Published:** 2017-04-19

**Authors:** Mikayla F. A. Baxter, Ruben Merino-Guzman, Juan D. Latorre, Brittany D. Mahaffey, Yichao Yang, Kyle D. Teague, Lucas E. Graham, Amanda D. Wolfenden, Xochitl Hernandez-Velasco, Lisa R. Bielke, Billy M. Hargis, Guillermo Tellez

**Affiliations:** ^1^Department of Poultry Science, University of Arkansas, Fayetteville, AR, USA; ^2^Facultad de Medicina Veterinaria y Zootecnia, Universidad Nacional Autónoma de México, Mexico City, Mexico; ^3^Department of Animal Science, The Ohio State University, Columbus, OH, USA

**Keywords:** broiler chickens, enteric inflammation, fluorescein isothiocyanate dextran, feed restriction, gut permeability

## Abstract

Fluorescein isothiocyanate dextran (FITC-d) is a 3–5 kDa marker used to measure tight junction permeability. We have previously shown that intestinal barrier function can be adversely affected by stress, poorly digested diets, or feed restriction (FR), resulting in increased intestinal inflammation-associated permeability. However, further optimization adjustments of the current FITC-d methodology are possible to enhance precision and efficacy of results in future. The objective of the present study was to optimize our current model to obtain a larger difference between control and treated groups, by optimizing the FITC-d measurement as a biomarker in a 24-h FR model to induce gut permeability in broiler chickens. One *in vitro* and four *in vivo* independent experiments were conducted. The results of the present study suggest that by increasing the dose of FITC-d (8.32 versus 4.16 mg/kg); shortening the collection time of blood samples (1 versus 2.5 h); using a pool of non-FITC-d serum as a blank, compared to previously used PBS; adding a standard curve to set a limit of detection and modifying the software’s optimal sensitivity value, it was possible to obtain more consistent and reliable results.

## Introduction

**Figure 1:**
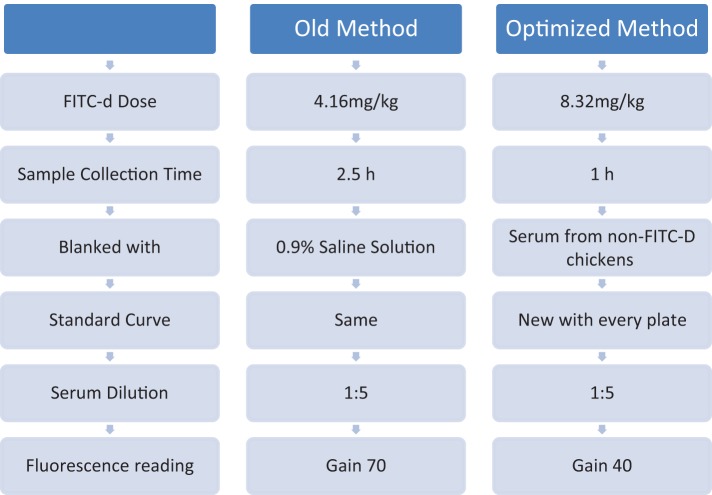
**Comparing old versus optimized methodology**.

Intestinal epithelial cells are not only responsible for digestion, secretion, and absorption but also act as a physical barrier separating external environmental agents from the internal host environment. In addition to preventing the entry of harmful intraluminal microorganisms, antigens, and toxins, this barrier increases the bodies’ tolerance to nutrients, water, and electrolytes (1–3). Microbes that live inside and/or on animals outnumber the animals’ actual somatic and germ cells by an estimated 10-fold (4). Hence, any alterations in gut permeability are associated with bacterial translocation to the portal and/or systemic circulation leading to systemic bacterial infections (5, 6). Consequently, our laboratory has recently developed several models to induce intestinal inflammation in poultry. Those models include high non-starch polysaccharides diets (7, 8); dexamethasone (9); dextran sodium sulfate (DSS) (10, 11); and 24-h feed restriction (FR) (12, 13). In the above models, inflammation causes disruption of the epithelial tight junctions (TJs) increasing bacterial translocation and leakage of serum fluorescein isothiocyanate dextran (FITC-d). FITC-d is a large molecule (3–5 kDa) which under normal conditions is not able to cross the epithelial barrier (14). However, during intestinal inflammation, the TJs are disrupted allowing the FITC-d molecule to enter circulation. Previous results from our laboratory have demonstrated that in poultry, chemically induced disruption of TJs with DSS (10) increases transmucosal permeability as seen by elevated serum levels of FITC-d (11). On the other hand, recently, we have shown that dietary inclusion of a *Bacillus*-based direct-fed microbial ameliorated the adverse gut permeability inflammatory effects related to utilization of rye-based diets in turkeys and in broiler chickens (15, 16). We have previously shown that FITC-d can be used as a biomarker for intestinal barrier function (7–12). However, further optimization adjustments of the current FITC-d methodology are possible to enhance precision and efficacy of results in future studies as can be observed in Figure 1 and Table 5. The objective of the present study was to optimize our current FITC-d model to obtain a larger difference between control and treated groups, using our 24-h FR model to induce gut permeability in broiler chickens.

## Materials and Methods

### Fluorescein Isothiocyanate Dextran

Fluorescein isothiocyanate dextran (MW 3–5 KDa; Sigma Aldrich Co., St. Louis, MO, USA) was used as a marker of paracellular transport and mucosal barrier dysfunction.

### *In Vitro* Evaluation of Different Fluorescence Gain Using Blank Chicken Sera from Chickens without FITC-d

Unlike absorbance assays where the gain on the plate reader is fixed and not user changeable, fluorescence assays have varying concentration ranges and require the gain on the photomultiplier to be adjusted. In this *in vitro* experiment, the following formula was used to predict the relative fluorescence units when changing the gain:

$$\text{Estimate of RFU at new gain setting}={\text{(new PMT gain/old PMT gain)}}^{7.3}\times \text{RFU at old PMT gain.}$$

To determine if fluorescence changes with varying gain, blank chicken sera and 0.9% saline were compared. Non-FITC-d chicken sera was diluted 1:5 in 0.9% saline onto black 96-well fluorescent plates and measured from gain 40 to gain 80 with continuous increments of 10. Non-FITC-d sera were also used to develop a standard curve adapted for every plate using six two-fold serial dilutions from the highest value 6,400 ng/mL until it reach 0 ng/mL (Table 1).

**Table 1:** **Evaluation of different fluorescence gain using blank chicken sera, from chickens without fluorescein isothiocyanate dextran (FITC-d), versus 0.9% saline solution**.

	Gain 40	Gain 50	Gain 60	Gain 70	Gain 80
0.9% saline solution	1.0 ± 0.27^b^	0.75 ± 0.31^b^	12.6 ± 0.18^b^	37.9 ± 0.74^b^	93.1 ± 1.3^b^
Blank sera	1.6 ± 0.11^a^	1.1 ± 0.10^a^	20.6 ± 0.38^a^	59.8 ± 1.1^a^	255.6 ± 6.6^a^

*Non-FITC-d sera was diluted 1:5 in 0.9% saline onto black 96-well fluorescent plates and measured from gain 40 to 80*.

*^a,b^Superscripts within columns indicate significant difference at P < 0.05*.

*Data are expressed as mean ± SE. n = 20 birds/treatment*.

### Experimental Animals

Four *in vivo* experiments were conducted to determine the optimal procedure for using FITC-d as a biomarker for intestinal permeability. In all trials, broiler chickens were obtained from a primary breeder company and all experiments were conducted in battery cages in a controlled age-appropriate environment.

### FR Model

In all experiments, intestinal permeability was induced using FR as previously published (12, 13). Chickens were randomly assigned to each experimental group and had unrestricted access to feed and water from 1 to 10 days of age. Beginning at 10 days, chickens in control FITC-d groups were allowed to continue with *ad libitum* access to feed, while chickens in FR FITC-d groups were subjected to 24 h of FR. Concentration of FITC-d was given based on group body weight; therefore, groups were weighed the day before FR began. At 11 days of age, chickens in all groups were given an appropriate dose of FITC-d by oral gavage for each experiment. After 1 h, or 2.5 h respectively, chickens were euthanized with CO_2_ asphyxiation. Blood samples were collected from the femoral vein to quantify levels of FITC-d.

### Serum Determination of FITC-d

In all experiments, blood was centrifuged (1,000 × *g* for 15 min) to separate the serum from the red blood cells. FITC-d levels of diluted sera were measured at excitation wavelength of 485 nm and emission wavelength of 528 nm (Synergy HT, Multi-mode microplate reader, BioTek Instruments, Inc., VT, USA). Fluorescence measurements were then compared to a standard curve with known FITC-d concentrations (old method) or non-FITC-d sera obtain from each independent experiment, respectively, to develop a standard curve as described in the *in vitro* methods.

### Experimental Designs

#### Experiment 1: Comparing Two Dilution Methods on Serum FITC-d Read at Gain 70 in a 24-h FR Model

Eighty chickens were randomly assigned to one of four groups (*n* = 20/group): (1) control no FITC-d; (2) FR no FITC-d; (3) control FITC-d 4.16 mg/kg; and (4) FR FITC-d 4.16 mg/kg. Control groups had *ad libitum* access to feed, meanwhile FR groups were feed restricted for 24 h before sampling. Serum was diluted at 1:5 or 1:10 to determine if a higher dilution factor would eliminate some of the background fluorescence. Readings were performed with a gain 70.

#### Experiment 2: Comparing Two Sampling Collection Times and Different Gain Readings of Serum FITC-d in a 24-h FR Model

In this experiment, all chickens received FITC-d (4.16 mg/kg) and samples were collected at 1 or 2.5 h post FITC-d administration. Eighty chickens were randomly assigned to one of four groups (*n* = 20/group): (1) control FITC-d collected 1 h post gavage; (2) FR FITC-d collected 1 h post-gavage; (3) control FITC-d collected 2.5 h post-gavage; and (4) FR FITC-d collected 2.5 h post-gavage. Control groups had *ad libitum* access to feed, meanwhile FR groups were feed restricted for 24 h before sampling. Serum was diluted at 1:5 and readings were done with gains 30, 35, 40, and 45.

#### Experiment 3: Comparing Collection Time of Serum FITC-d Diluted 1:5 and Read at Gain 40 in a 24-h FR Model

In this experiment, all chickens received FITC-d (8.32 mg/kg) and samples were collected at 1 or 2.5 h post FITC-d administration. Eighty chickens were randomly assigned to one of four groups (*n* = 20/group): (1) control FITC-d collected 1 h post-gavage; (2) FR FITC-d collected 1 h post-gavage; (3) control FITC-d collected 2.5 h post-gavage; and (4) FR FITC-d collected 2.5 h post-gavage. Control groups had *ad libitum* access to feed, meanwhile FR groups were feed restricted for 24 h before sampling. Serum was diluted at 1:5 and readings were done using gain 40.

#### Experiment 4: Comparing the Old Method versus Optimized Method of Serum FITC-d in a 24-h FR Model

The objective of this experiment was to compare our previous FITC-d method to the new optimized FITC-d method. Eighty chickens were randomly assigned to one of four groups (*n* = 20/group): (1) control FITC-d (4.16 mg/kg) collected 2.5 h post-gavage; (2) FR FITC-d (4.16 mg/kg) collected 2.5 h post-gavage; (3) control FITC-d (8.32 mg/kg) collected 1 h post-gavage; and (4) FR FITC-d (8.32 mg/kg) collected 1 h post-gavage. In the old method, serum was diluted 1:5, fluorescence measurements were quantified using an equation from a previously determined standard curve with known FITC-d concentrations using 0.9% saline solution as a blank and measuring samples at gain 70. In the optimized method, serum from non-FITC-d chickens was obtained, to be used as a blank. Additionally, for each plate, a standard curve was adapted diluting known concentrations of FITC-d in the 1:5 diluted blank sera as described above in the *in vitro* method. All serum samples were also diluted 1:5 for fluorescence reading at gain 40 (Table 5).

### Statistical Analysis

All data were subjected to Analysis of Variance as a completely randomized design using the General Linear Models procedure of SAS (17). In all trials, data are expressed as mean ± standard error. Significant differences among the means were determined by using Tukey’s multiple-range test at *P* < 0.05.

## Results

The results of the *in vitro* evaluation of different fluorescence gains using blank chicken sera, from chickens without FITC-d, versus 0.9% saline solution are summarized in Table 1. There was a significant difference between blank sera and 0.9% saline solution at each of the gains measured (40, 50, 60, 70, 80) (Table 1). This indicates that blank sera has a higher amount of fluorescence activity than 0.9% saline and is affected by the gain. Table 2 illustrates the results from Experiment 1 comparing two serum dilution methods on serum FITC-d (4.16 mg/kg) read at gain 70 in a 24-h FR model. In this study, using the same sera, samples were diluted 1:5 and 1:10 to determine if a higher dilution factor would eliminate some of the background fluorescence. A significant reduction in the background fluorescence was observed in all samples diluted at 1:10 (*P* < 0.05). Interestingly, serum samples from FR chickens treated with FITC-d diluted at 1:5 or 1:10 showed significantly higher amounts of serum FITC-d concentration when compared with control chickens.

**Table 2:** **Comparing two serum dilution methods on serum fluorescein isothiocyanate dextran (FITC-d) (4.16 mg/kg) read at gain 70 in a 24-h feed restriction (FR) model to induce gut permeability in broiler chickens (Experiment 1)**.

Experimental group	Serum FITC-d (ng/mL) Diluted 1:5	Serum FITC-d (ng/mL) Diluted 1:10
Control no FITC-d	7.7 ± 2.7^b,y^	1.0 ± 0.9^b,z^
FR no FITC-d	11.4 ± 3.4^b,y^	2.5 ± 1.6^b,z^
Control FITC-d	9.1 ± 2.8^b,y^	2.5 ± 1.2^b,z^
FR FITC-d	23.1 ± 4.3^a,y^	16.8 ± 3.1^a,z^

*^a,b^Superscripts within columns indicate significant difference at P < 0.05*.

*^y,z^Superscripts within rows indicate significant difference at P < 0.05*.

*Data are expressed as mean ± SE. n = 20 birds/treatment. In both comparisons, blanked serum was used to make a standard curve with every plate*.

Results from Experiment 2 comparing two sampling collection times and different gain readings of serum FITC-d (4.16 mg/kg) in a 24-h FR model are summarized in Table 3. Collecting the blood samples 1 h post FITC-d gavage not only showed significant increases in serum FITC-d concentration in chickens that received FR when compared with control chickens at all four gain readings but also the window of differences between feed restricted and control broilers were more evident when compared with serum collected at 2.5 h (Table 3).

**Table 3:** **Comparing two sampling collection times and different gain readings of serum fluorescein isothiocyanate dextran (FITC-d) (4.16 mg/kg) in a 24-h feed restriction (FR) model to induce gut permeability in broiler chickens (Experiment 2)**.

Experimental group	Serum FITC-d (ng/mL) Gain 30	Serum FITC-d (ng/mL) Gain 35	Serum FITC-d (ng/mL) Gain 40	Serum FITC-d(ng/mL) Gain 45
Control FITC-d 1 h	0.00 ± 0.0.00^c,z^	61.0 ± 21.4^c,y^	49.6 ± 16.2^c,y^	56.3 ± 16.2^c,y^
FR FITC-d 1 h	207.5 ± 69.9^b,y^	284.4 ± 37.7^b,x^	185.6 ± 14.5^a,z^	177.1 ± 13.9^a,z^
Control FITC-d 2.5 h	390.8 ± 84.4^a,x^	257.0 ± 25.0^b,y^	118.9 ± 11.0^b,z^	95.4 ± 8.8^b,z^
FR FITC-d 2.5 h	468.1 ± 75.7^a,x^	336.5 ± 41.9^a,y^	191.3 ± 24.0^a,z^	153.3 ± 19.4^a,z^

*^a,b,c^Superscripts within columns indicate significant difference at P < 0.05*.

*^x,y,z^Superscripts within rows indicate significant difference at P < 0.05*.

*Data are expressed as mean ± SE. n = 20 birds/treatment. Serum was diluted 1:5. Blanked serum was used to make a standard curve with every plate*.

Table 4 displays the results from Experiment 3, comparing collection time of serum FITC-d doubling the dose of FITC-d (8.32 mg/kg). Serum was diluted at 1:5 and read at gain 40 in a 24-h FR model. These results confirmed and extended the results of Experiment 2. Sample collection time gives a stronger reading of serum FITC-d in FR chickens when is performed 1 h after FITC-d oral administration when compared with 2.5 h (Table 4).

**Table 4:** **Comparing collection times of serum fluorescein isothiocyanate dextran (FITC-d) (8.32 mg/kg) diluted 1:5 and read at gain 40 in a 24-h feed restriction (FR) model to induce gut permeability in broiler chickens (Experiment 3)**.

Experimental group	Serum FITC-d (ng/mL)
Control 1 h	78.7 ± 9.4^c^
FR 1 h	136. 5 ± 7.3^a^
Control 2.5 h	67.1 ± 7.9^c^
FR 2.5 h	112.4 ± 6.5^b^

*^a,b,c^Superscripts within columns indicate significant difference at P < 0.05*.

*Data are expressed as mean ± SE. n = 20 birds/treatment*.

The results from Experiment 4, comparing old method versus optimized method of serum FITC-d in a 24-h FR model are summarized in Figure 1 and Table 5. In the old method, chickens received 4.16 mg/kg FITC-d, serum samples were collected 2.5 h post gavage, samples were diluted 1:5 and fluorescence was measured using a previously determined standard curve, 0.9% saline solution was used as a blank and measured at gain 70. No significant differences were observed between control and FR chickens. In contrast, in the optimized method, chickens receiving 8.32 mg/kg FITC-d, serum samples were collected 1 h post gavage, were diluted 1:5, non-FITC-d serum was used as a blank, a standard curve was developed for each plate and a reading of gain 40, showed significant differences between control and FR chickens (Figure 1; Table 5).

**Table 5:** **Comparing old method versus optimized method of serum fluorescein isothiocyanate dextran (FITC-d) in a 24-h feed restriction (FR) model to induce gut permeability in broiler chickens (Experiment 4)**.

Experimental group	Serum FITC-d (ng/mL) Old method	Serum FITC-d (ng/mL) Optimized method
Control	306.0 ± 41.1^a,y^	101.8 ± 36.0^b,z^
FR	388.0 ± 28.0^a,z^	397.3 ± 22.1^a,z^
FITC-d dose (mg/kg)	4.16	8.32
Sample collection time (h)	2.5	1
Blanked with	0.9% saline solution	Serum from non-FITC-d chickens
Standard curve	Same	New with every plate
Serum dilution	1:5	1:5
Fluorescence reading	Gain 70	Gain 40

*^a,b^Superscripts within columns indicate significant difference at P < 0.05*.

*^y,z^Superscripts within rows indicate significant difference at P < 0.05*.

*Data are expressed as mean ± SE. n = 20 birds/treatment*.

## Discussion

Stress is known to affect gastrointestinal tract (GIT) homeostasis by altering gut motility, permeability, as well as alterations in ion, fluid, and mucus secretion and absorption (18–21). Several investigators have reported that acute or chronic stress modifies gut permeability associated with a temporary redistribution of TJ proteins (22–25). Some of these alterations are linked to mast cells in the brain–gut axis which secrete several neurotransmitters and pro inflammatory cytokines, with profound effects on GIT physiology (26–28). Another hormone that increases during acute or chronic stress is corticotrophin-releasing factor, which increases intestinal paracellular permeability *via* mast cell-dependent release of TNF-α and proteases (29–31). Moreover, excessive cortisol may lead to GIT disturbances, opportunistic infections, and impaired wound healing (32–34). Due to intensive selection, modern chickens are the most efficient meat-producing animals because of their fast growth, supported by a virtually unlimited voluntary feed intake. However, these features also cause many problems in breeder hens because of the negative correlation between muscle growth and reproduction effectiveness. Hence, commercial restricted feeding programs in broiler breeders have been implemented, with negative effects on welfare and health, as birds are continuously hungry (35). Previous research in poultry has shown that FR increases plasma levels of corticosterone causing disruption of gut barrier integrity, systemic, and local inflammation (36–39). Similarly, we have previously shown in poultry that intestinal barrier function can be adversely affected by stress, poorly digested diets (7, 8), or FR (12, 13), resulting in increased intestinal inflammation-associated permeability. In those studies, we have described a correlation of liver bacterial translocation and serum concentrations of FITC-d as markers used to measure TJ permeability. FITC-d is a 3–5 kDa marker used to measure TJ permeability in chickens using enteric inflammation models. However, inconsistent results obtained from unpublished data suggested that current FITC-d methodology required further optimization. FITC-d has also been reported to be a viable method to measure enteric leakage in the murine model (40). However, they used a different methodology. Therefore, FITC-d methodology may vary with the animal model and this should be taken into consideration when using it to measure gut permeability. The results of the present study suggest that by increasing the dose of FITC-d (8.32 versus 4.16 mg/kg); shortening the collection time of the blood (1 versus 2.5 h); using a pool of non-FITC-d serum as a blank, compared to previously used 0.9% saline; generating a standard curve with every plate to set a limit of detection and modifying the software’s optimal sensitivity value, it is possible to obtain more consistent and reliable results when measuring gut leakage in poultry.

## Ethics Statement

All animal handling procedures were in compliance with Institutional Animal Care and Use Committee at the University of Arkansas.

## Author Contributions

MB, RM-G, JL, and GT contributed to the overall study design and supervised all research. BM, YY, KT, LG, and AW carried out the experiments and acquisition of data. MB and GT drafted and revised the first version of the manuscript. JL and GT analyzed the data. LB, XH-V, BH, and GT drafted the article and revised it critically for important intellectual content. XH-V and GT were responsible for the final editing of the manuscript. All the authors reviewed and finally approved the manuscript.

## Conflict of Interest Statement

The authors declare that the research was conducted in the absence of any commercial or financial relationships that could be construed as a potential conflict of interest.
